# Supplementary material for: Involvement of MicroRNA-1-FAM83A Axis Dysfunction in the Growth and Motility of Lung Cancer Cells
Source: Int J Mol Sci. 2020 Nov 22;21(22):8833. doi: 10.3390/ijms21228833 (PMC7700477; doi:10.3390/ijms21228833)
Supplement: Supplementary file 1 [file ijms-21-08833-s001.pdf]

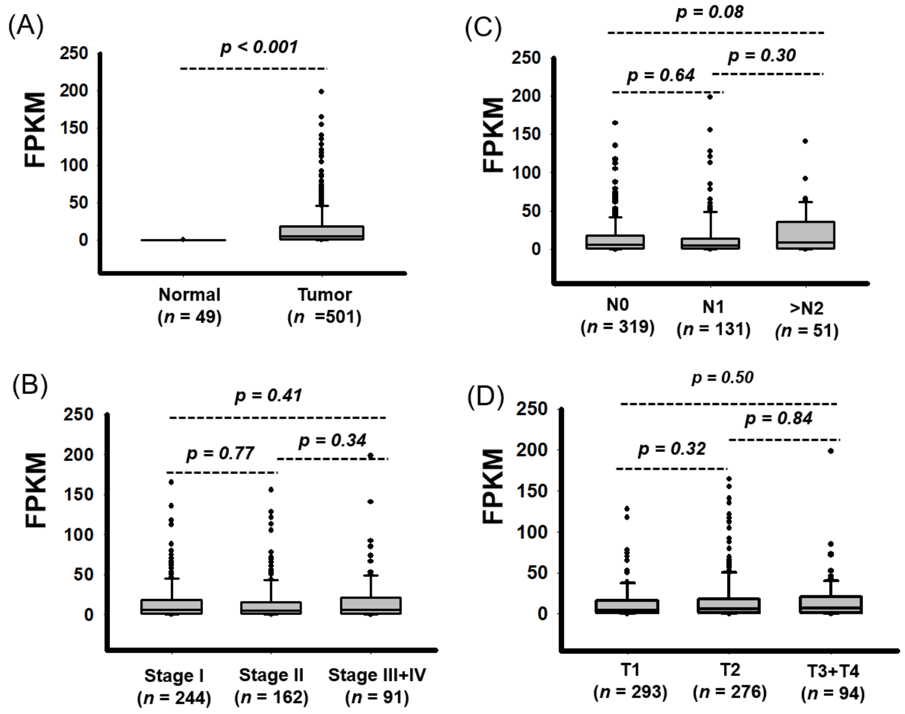

Figure S1. Association of *FAM83A* Expression With Poor Prognosis in LUSC. (**A**) *FAM83A* expression in LUSC compared with in corresponding adjacent normal tissues. (**B**) *FAM83A* expression in LUSC by pathological stage. (**C**) *FAM83A* expression in LUSC by lymph node metastasis status. (**D**) *FAM83A* expression in LUAD by T stage.

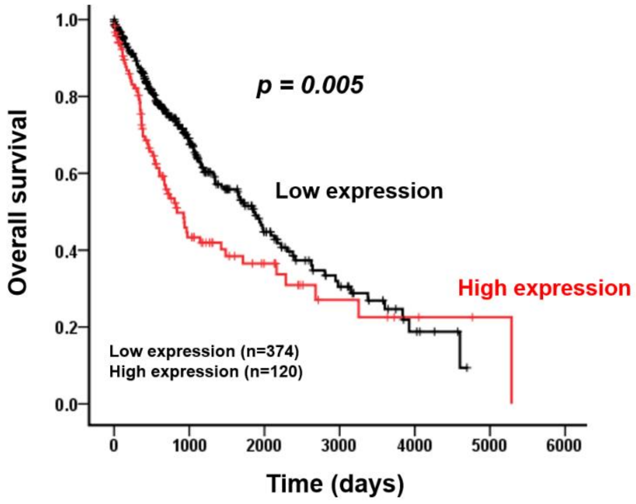

Figure S2. Kaplan-Meier Survival Curves Showing the Effect of *FAM83A* Expression on Overall Survival in LUSC.

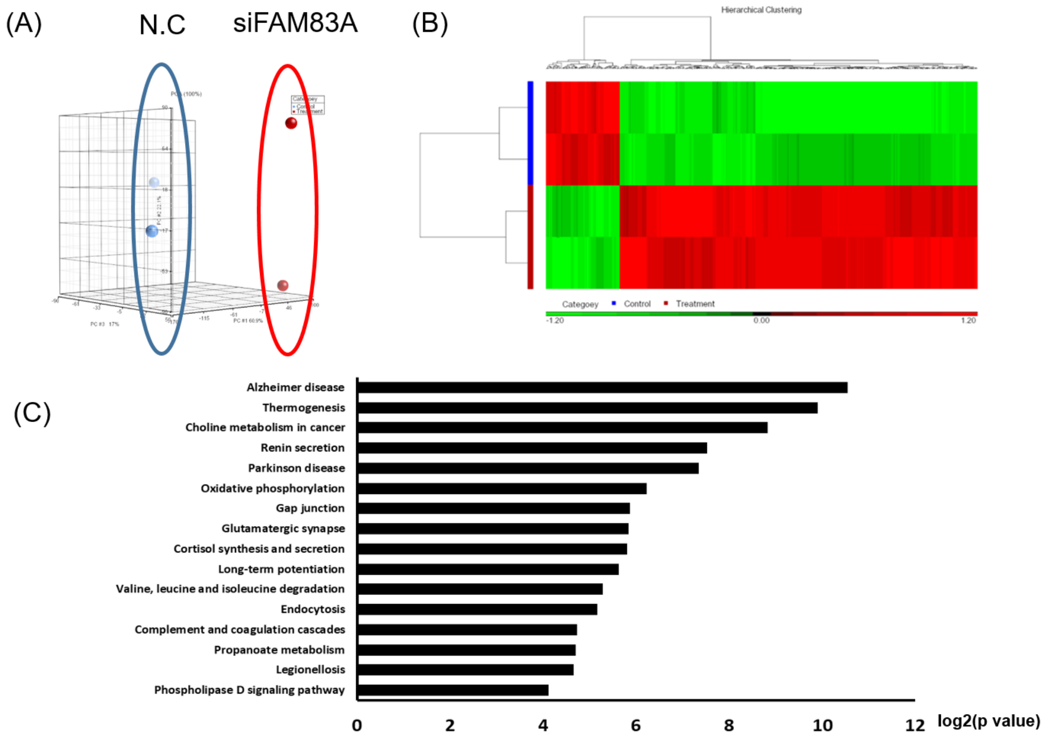

Figure S3. Identification of Downstream genes of *FAM83A* in A549 Cells through Microarray Data Analysis. After *FAM83A* knockdown for 48 h, the total RNA was extracted and subjected to transcriptome profiling through microarray data analysis. Differentially expressed genes were selected according to 2 criteria: changes >1.5-fold or <−1.5 fold and $p < .05$. (**A**) Four RNA transcriptomes were analyzed using principal coordinate analysis. (**B**) Heat maps showing the differentially expressed genes. (**C**) Pathway enrichment analysis was performed on the differentially expressed genes. The signaling pathways are on the Y-axis. The X-axis is $\log_2 p$-scaled.

Table S1. The gene candidates differentially expressed in lung cancer with advance stage.

Gene_symbol	Gene Symbol	Stage I + II(mean)	Stage III + IV (mean)	p value
ENSG00000205362	MT1A	3.0	12.6	0.028
ENSG00000123999	INHA	4.7	15.4	0.012
ENSG00000146374	RSPO3	2.3	7.3	0.013
ENSG00000188505	NCCRP1	3.3	8.6	0.025
ENSG00000111432	FZD10	1.9	4.4	0.050
ENSG00000120211	INSL4	2.1	4.9	0.044
ENSG00000019186	CYP24A1	20.5	46.0	0.015
ENSG00000023839	ABCC2	2.9	6.4	0.015
ENSG00000107984	DKK1	6.9	14.2	0.019
ENSG00000104140	RHOV	16.0	32.7	0.005
ENSG00000147689	FAM83A	20.7	41.7	0.002
ENSG00000152377	SPOCK1	2.3	4.5	0.027
**Downregulation**	**Gene Symbol**	**Stage I + II(mean)**	**Stage III + IV (mean)**	**p value**
ENSG00000184357	H1-5	7.3	0.22	0.019
ENSG00000197061	H4C3	6.7	0.20	0.002
ENSG00000277157	H4C4	5.9	0.23	0.021
ENSG00000168631	MUCL3	18.0	1.10	0.007
ENSG00000274267	HIST1H3B	5.2	0.33	0.008
ENSG00000168298	H1-4	10.1	0.67	0.001
ENSG00000276966	H4C5	9.5	0.74	0.002
ENSG00000124575	H1-3	6.1	0.51	0.005
ENSG00000136110	CNMD	12.5	1.45	0.028
ENSG00000184956	MUC6	12.3	2.26	0.032
ENSG00000184260	H2AC20	7.0	1.71	0.001
ENSG00000196866	H2AC7	4.1	1.13	0.036
ENSG00000166961	MS4A15	12.7	3.62	0.000
ENSG00000149021	SCGB1A1	355.3	109.93	0.001
ENSG00000275713	H2BC9	4.6	1.53	0.021
ENSG00000196747	H2AC13	3.9	1.30	0.016
ENSG00000166448	TMEM130	4.9	1.83	0.000
ENSG00000161055	SCGB3A1	624.8	244.48	0.037
ENSG00000091583	APOH	9.3	3.89	0.047
ENSG00000273802	H2BC8	6.1	2.69	0.010
ENSG00000277075	H2AC8	7.0	3.14	0.022
ENSG00000171246	NPTX1	6.1	2.77	0.020
ENSG00000197409	H3C4	3.7	1.72	0.007
ENSG00000174946	GPR171	4.1	1.91	0.001
ENSG00000156738	MS4A1	3.7	1.79	0.001
ENSG00000181617	FDCSP	9.8	4.81	0.005

Table S2. Correlation of FAM83A expression with clinicopathological characteristics of 500 lung squamous cell carcinoma patients (LUSC).

Variables	FAM83A (*n* = 500)			
	**No. (%)**	**Mean ± SD**	**Median**	***p*-value**
**Pathology stage**				
I	244 (48.8)	15.35 ± 24.42	5.88	0.450[a]
II	163 (32.6)	14.54 ± 25.43	4.95	
III+ IV	93 (18.6)	18.70 ± 30.66	6.21	
**pT stage**				
T1	114 (22.8)	13.59 ± 22.34	4.50	0.600[a]
T2	292 (58.4)	16.49 ± 27.36	5.52	
T3 + T4	94 (18.8)	15.85 ± 25.91	7.23	
**pN stage** (n = 499)				
N0	324 (64.8)	15.04 ± 23.44	5.69	0.573[a]
N1	131 (26.2)	15.96 ± 30.83	5.09	
>N2	45 (9.0)	19.37 ± 28.50	6.57	
**pM stage**				
M0	493 (98.6)	15.69 ± 26.10	5.58	0.898[b]
M1	7 (1.4)	16.97 ± 18.39	9.92	

[a] *p*-value were estimated by one-way ANOVA test. [b] *p*-value were estimated by Student's *t* test.

Table S3. Univariate and multivariate Cox's regression analysis of FAM83A expression for overall survival of 494 patients with LUSC.

**Characteristic**	No. (%)	OS			
		CHR (95% CI)	*p*-value	AHR (95% CI)	*p*-value
FAM83A	(n = 494)				
Low	374 (75.7)	1.00		1.00	
High	120 (24.3)	1.52 (1.14–2.05)	0.005	1.49 (1.11–2.00)	0.008

Abbreviation: DSS, disease-specific survival; DFS, disease-free survival; CHR, crude hazard ratio; AHR, adjusted hazard ratio. AHR were adjusted for AJCC pathological stage (II,III and IV VS. I).
